# Application and Clinical Effectiveness of Antibiotic-Loaded Bone Cement to Promote Soft Tissue Granulation in the Treatment of Neuropathic Diabetic Foot Ulcers Complicated by Osteomyelitis: A Randomized Controlled Trial

**DOI:** 10.1155/2021/9911072

**Published:** 2021-07-14

**Authors:** Regis Ernest Mendame Ehya, Hao Zhang, Baiwen Qi, Aixi Yu

**Affiliations:** Department of Orthopedics Trauma and Microsurgery, Zhongnan Hospital of Wuhan University, Wuhan, 430071 Hubei, China

## Abstract

This study explored the clinical effectiveness of antibiotic-loaded bone cement on primary treatment of diabetic foot infection. This is a randomized controlled study, including thirty-six patients with diabetic foot ulcer complicated by osteomyelitis who had undergone treatment between May 2018 and December 2019. Patients were randomly divided into control group (group A) and study group (group B). Patients in the intervention group received antibiotic-loaded bone cement repair as primary treatment, while patients in the control group received conventional vacuum sealing draining treatment. Clinical endpoints were assessed and compared between the two groups, including wound healing time, wound bacterial conversion, NRS pain score, number of wound dressing changes, and average hospitalization time. All patients were followed up for a period of 12 months after discharge. Results show that compared with the control group, patients in the study group had significant difference in the number of patients for baseline pathogens eradication, short NRS pain score, hospital length of stay and cost, wound surface reduction, healing time, low rate of complications, and infection recurrence. Based on the findings, we conclude that antibiotic-loaded bone cement can be used for treatment of wound in patient with diabetic foot infection. It can help to control wound infections, shorten hospital length of stay, reduce medical cost, and relieve both doctors' and patients' burden. The application of antibiotic-loaded bone cement is suitable for diabetic wound with soft tissue infection or osteomyelitis.

## 1. Introduction

Diabetes mellitus (DM) is a pathology that affects millions of people worldwide, and the global prevalence has increased rapidly in the last thirty years. This trend is expected to continue increasing in the future from the current 5.1% to 7.7% in 2030 [1]. Diabetes-related foot complications have been identified as the single most common cause of morbidity among diabetic patients [2]. Diabetic foot complications have a prevalence of up to 25% and are the main reason for hospitalization and amputation in people with diabetes [3]. Up to 15% of all diabetic patients will be affected by a foot ulcer, and the recurrence of such ulcers is seen in over 70% of patients within 5 years [4, 5]. Principal causes of diabetic foot infections are vascular stenosis and occlusion, nerve axis mutation, and demyelination caused by tissue ischemia and hypoxia, hypoesthesia, foot tissue necrosis, and local infection. Severe cases often require amputation and even lead to death; this represents a serious threat to patients' health [6, 7]. While conventional treatment of neuropathic foot ulcers is usually done through offloading, debridement, and systemic dressing, ischemic ulcers often require antibiotherapy and surgery [4]. In this study, patients with neuropathic diabetic foot ulcers complicated by osteomyelitis were applied an antibiotic-loaded bone cement (ALBC) on the defect resulting from the surgical debridement of necrotized tissues to treat diabetic foot infection. We found that the application of antibiotic-loaded bone cement in the treatment of DFU could achieve a satisfying medical outcome.

## 2. Methods and Subjects

### 2.1. Study Design and Study Population

This is a randomized, monocentric, and controlled study conducted in the Department of Orthopedics Trauma and Microsurgery of Wuhan University, Zhongnan Hospital. We clinically assess the application and effectiveness of a bone cement loaded with specific antibiotic (according to the antibiotic sensitivity test results) in patients with type-2 diabetes mellitus in whom foot ulceration had been diagnosed, between 1 May 2018 and 31 December 2019.

The study included 36 patients with diabetic neuropathy, comprising 24 men and 12 women. Patients age ranged between 35 and 85 years old, and all patients were followed up to 12 months. Ulcers were classified as grades III and IV according to the Meggitt-Wagner system [2]. Patients were enrolled into the study if they met the following inclusion criteria: age > 18 years; neuropathic diabetic foot ulcers complicated by osteomyelitis; able to attend clinical visit during follow-up period; and gave written consent for inclusion in the study. Patients with neoplasms, specific infections, or wound infections not related to DM [8, 9]; patients with antibiotic allergies or who have received antibiotics 1 week prior hospital admission [10]; and those who for whatever reason refused to participate in the study were excluded. Patient age, sex, weight, height, body mass index (BMI), type of diabetes, HbA1c, ankle brachial index (ABI), and site of ulceration were recorded (Table 1). All patients received insulin therapy aiming at fasting serum glucose levels below 6 mmol/l.

### 2.2. Measurements

The peripheral diabetic neuropathy was evaluated by measuring the vibration perception threshold with the calibrated Rydel-Seiffer tuning fork [11]. Ankle brachial indexes were calculated by the pressure at the ankle by the brachial pressure. Ulcer healing was assessed by planimetric measurement of the wound area (cm^2^) using a foil with millimeter scales, which was laid on the ulcer every second week. The method of wound area size and healing time assessments was used as previously described by Zimny et al. [12]. The wound radius reduction is calculated from the virtual radius of the wound by the equation $R=\sqrt{A/\pi }$ and the healing time of the wound (*R* is the radius, *A* is the planimetric wound area (cm^2^), and *π* the constant 3.14).

### 2.3. Clinical Data and Randomization

**Figure 1:**
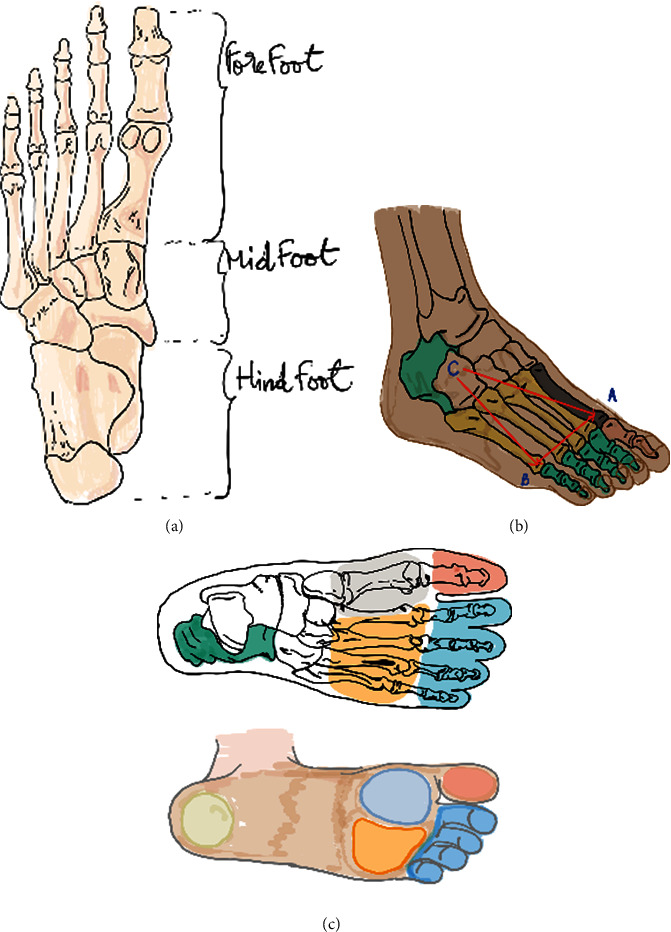
Schematic drawing of the foot regions. (a) Dorsal view, (b) foot weight-bearing tripod, and (c) foot areas of high risk for ulceration.

During the study enrolment period, 36 patients with type-2 diabetes mellitus in whom foot ulceration suggestive to osteomyelitis (probe to bone and positive radiological features) had been diagnosed and did meet the inclusion criteria were randomly divided into two groups—the control group and study group. The control group B was treated with vacuum sailing drainage (VSD) treatment, while the study group A received antibiotic-loaded bone cement (ALBC) treatment. Eighteen patients were randomly divided to each group. Patients' age range was 35-85 years old, with an infected area of 5-28 cm^2^. The most common sites of diabetic foot infections were as follows: 18 patients in forefoot (77.8%), 11 patients in the midfoot (13.3%), and 7 patients (8.9%) in the hindfoot (Figure 1, Table 1). While patient from control group received a traditional treatment, those from study group were treated with an antibiotic-loaded bone cement.

### 2.4. Treatment

#### 2.4.1. Preoperative Treatment

Before operation, the lesions of the two groups were examined by plain film X-rays and magnetic resonance imaging (MRI) especially after a prolonged infection, blood leukocytes, and C-reactive protein levels were monitored. Wound secretions and bacterial cultures of the diseased tissue and drug sensitivity tests were carried out. Both groups received comprehensive medical treatment: quitting smoking and alcohol, controlling blood sugar, and applying vasodilator drugs.

#### 2.4.2. Bacterial Culture and Antibiotic Selection

Microbial data analysis based on culture at baseline showed that prevalent pathogens were *Pseudomonas aeruginosa*-positive in 11 cases (sensitive to cefoperazone plus sulbactam), *Escherichia coli*-positive in 4 cases (sensitive to gentamicin), and *Staphylococcus aureus*-positive in 21 cases (sensitive to vancomycin) (Table 2). Antibiotics were selected according to the results of bacterial culture and drug sensitivity of wound secretion and used following the antibiotic-laden polymethyl-methacrylate bone cement (Heraeus, Beijing Landmover Medical Company, Beijing, China). The ratio of various antibiotics was used as previously recorded by Liu et al. [13].

#### 2.4.3. Surgical Procedure and Postoperative Treatment

**Figure 2:**
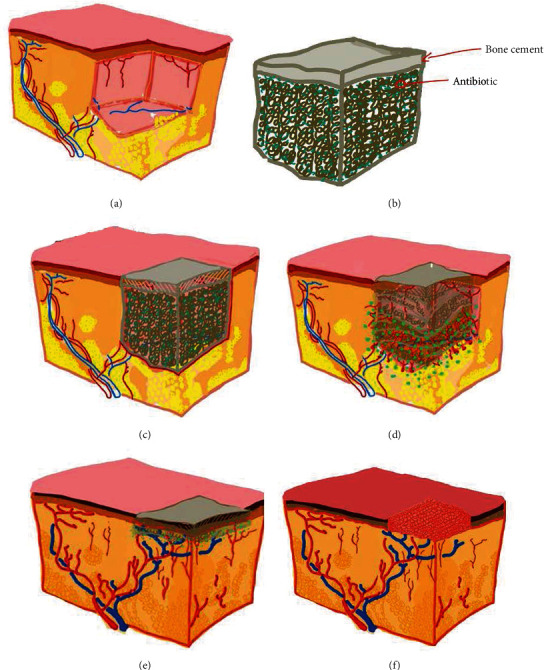
Schematic drawing of the antibiotic-loaded bone cement procedure. (a) Dermal defect. (b) Antibiotic-loaded bone cement. (c) Antibiotic-loaded bone cement was implanted into the defect site, and release of the antibiotic. (d) Fibroblast and vascular endothelial cells grow into the scaffolds from the wound base and surrounding tissues, forming a new capillary cell complex. (e) After 3 weeks, the bone cement has degraded from the initial size, leaving only a remaining coat at the wound surface, covering a good granulated soft tissue. (f) The defect tissue was regenerated, living option for sutures or split thickness skin coverage of the wound.

**Figure 3:**
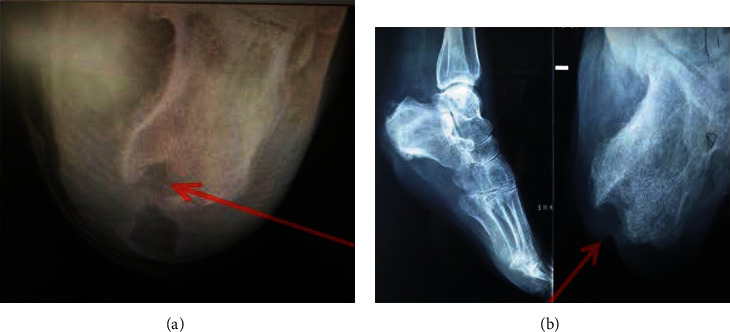
Clinical radiographies of diabetic heel ulceration (a) before surgery and (b) 12 weeks after surgery. The arrow head is pointed on the calcaneus.

**Figure 4:**
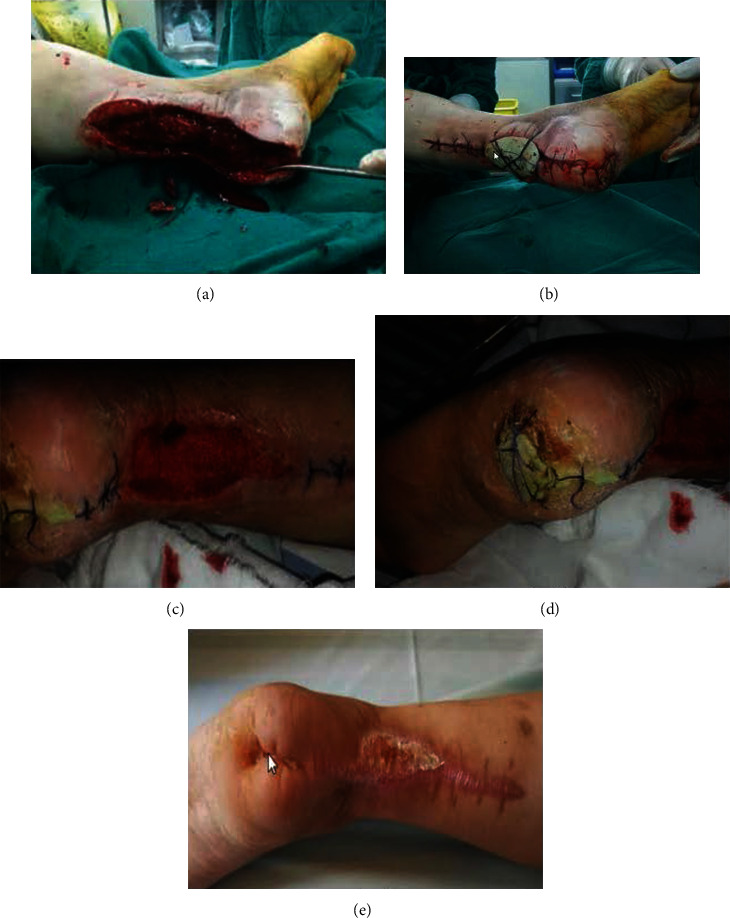
Diabetic heel ulceration. Diabetic heel ulceration (a) during surgery, (b) immediately after surgery, (c, d) 3 weeks after surgery, and (e) 12 weeks after surgery.

A multidisciplinary team including orthopedic trauma surgeon, endocrinologist, microbiologists, and nurse treated all patients based on a collaborative care model [14]. According to the shape of the wound surface, antibiotic bone cement was used to cover the entire surface. Nevertheless, it is worthy of note the surgeon waited for the antibiotic-loaded bone cement-mixed body temperature to be significantly reduced, in order to prevent soft tissue being damaged by the heat, and then the wound surface was covered with sterile dressing (Figure 2). After complete examination and preoperative routine management (Figure 3(a)), the operation was performed. Debridement thoroughly according to conventional procedures was done to patients wounds in both groups [15]. The administration of the anesthesia was done under intraspinal. Patients from control group were thoroughly surgically treated according to the surgical technique previously described by Liu et al. [13]. In the study group A, after debridement of the wound and cleansing with normal saline and 1.5% volume fraction of hydrogen peroxide solution in turn, the specific antibiotic (vancomycin, cefoperazone, or gentamicin) powder and bone cement powder were mixed at a ratio of 3 : 40, and the monomer was added to make a paste (Figure 4).

Patient wound dressing in both groups was reexamined every day after surgery until hospital discharge. They were reexamined for bacterial culture of wound secretions, and blood leukocytes, C-reactive protein levels, and intravenous antibiotics were given after operation every three days. Patients in the study group changed their dressings routinely and used sterile gauze to absorb local powder exudates during dressing changes. If there were profuse secretions, the wound must be rinsed and then covered with new molded antibiotic-loaded bone cement mass. If there were no signs of inflammatory cell infiltration around the wound surface, the wound surface is partially induced to form a membrane, and the patients could be discharged from the hospital. Observation is performed at the outpatient clinic, and the dressing is changed once or twice a week until the wound surface presented a good granulated soft tissue or has completely healed. In the control group, debridement and replacement of vacuum sealing drainage dressing were performed once a week. When the wound infection in the control group B was totally controlled or considerably reduced towards eradication, the patient was discharged. And all the follow-up observation was performed at the outpatient clinic, where the dressing was changed until the wound surface has completely healed (Figure 3(b)).

#### 2.4.4. Definition of Some Clinical Outcomes

Ulceration with osteomyelitis was defined as having visible exposed bone, bone palpable with a blunt probe, or a deep ulcer persistent over a bony prominence and the presence of a soft tissue sinus with purulent discharge, confirmed by positive radiological features [6, 16]. We defined *healing* as the complete epithelialization of the ulcer and/or the surgical wound that was created while treating the infection [10]. *Healing time* was defined as the time in days from the date on which DFU patient received the treatment to the date of wound surface complete reepithelization or soft tissue granulation, complete healing. A novel episode of ulcer occurring during follow-up time, at the same site or nearby, was defined as *reulceration*. *Recurrence of osteomyelitis* was attributed to the appearance bone infections at the same or/and an adjacent site after healing of both the ulcer that was the point of entry of the infection and the surgical wound. *New osteomyelitis* was classified where new ulcer was complicated by bone infection [10].

### 2.5. Statistical Endpoints

The primary endpoint was the healing of the diabetic ulceration with no subsequent surgery. The secondary endpoints were mean wound healing time, hospital length of stay, numeric pain score, and complication rates between the two groups.

### 2.6. Statistical Analysis

The data were analyzed using SPSS software version 19.0 (IBM SPSS Statistics for Windows; Armonk, NY, IBM Corp., USA). Categorical variables were evaluated using Chi-square test or Fisher's exact test. Continuous variables were evaluated using either a *t*-test or the Mann–Whitney test, based on whether data distribution was normal or nonnormal. *p* < 0.05 was considered statistically significant.

### 2.7. Ethical Considerations

The study protocol abided by the Chinese government's ethical guidelines for clinical and case study research, and it was reviewed and approved by the ethics committee of Zhongnan Hospital. All procedures were undertaken in accordance with the ethical standards established by the institutional and national committees on human experimentation and in accordance with the Declaration of Helsinki. The informants were given oral and written information that the study was voluntary and that they had the right to withdraw their participation at any time without any explanation. Written informed consent was obtained from all subjects.

## 3. Results

### 3.1. Baseline Characteristics of Patients

Within the period of recruitment and implementation of the study, 36 patients with diabetic foot, including 24 males and 12 females aged between 45 and 75 years with an average age of 46.8 (±6.85) years, met the inclusion criteria. The mean age of the patients was 45 ± 6.3 years and 48 ± 5.1 years, for the control group and study group, respectively. The groups were composed of 11 males and 7 females, and 13 males and 5 females for control group B and study group A, respectively. The detailed clinical characteristics of the patients are summarized in Table 1. There was no statistical significant difference of age, gender, BMI, HbA1c, and ABI between the two groups (*p* > 0.05, Table 1). Nevertheless, there were mostly patients with type-2 diabetes mellitus in both groups.

### 3.2. Postoperative Pain Scores

The postoperative mean score of numeric rating scale for pain (NRS pain) reported for the study group was 1.4 ± 0.9, whereas that for the control group was 3.3 ± 1.4 (*p* < 0.01, Table 3).

### 3.3. Hospital Length of Stay and Cost

Hospital length of stay recorded in days for patients in control group was 29 ± 2 days, whereas patients randomized in the study group recorded a shorter hospital stay compared with the other group (25 ± 2 days). There was no statistical significance between the two groups (*p* > 0.05, Table 3).

The mean hospital cost was compared between the two groups. The average hospital cost recorded in the control group was significant and higher compared to the study group ($4100 ± 800 vs. $3700 ± 460; *p* < 0.001, Table 3).

### 3.4. Number of Dressing Change and Wound Complete Healing Time

The statistical analyses revealed that the study group had significantly fewer dressing renewals (*p* < 0.01, Table 3). The wound area (mean ± SD) of diabetic foot ulcer in the control group was 18 ± 10.5 cm^2^ at the beginning, and 3.2 ± 0.5 cm^2^ after 84 days (*p* < 0.05) (Table 4). The calculated wound radius decreased by 0.037 cm (95% confidence interval (CI), 0.033–0.041) per day. The average healing time was 101.7 (95% CI, 93–110) days. In the study group, the average wound area was 17.5 ± 11.0 at the start of the ulcer care, and 2.1 ± 0.5 cm^2^ after 12 weeks (*p* < 0.05). Daily wound radius reduction was calculated by 0.049 mm (95% CI, 0.043–0.057) with an average healing time of 79.4 (95% CI, 71–90) days. Statistical analyses showed that there was significant difference between the two groups in terms of healing time and wound radius reduction (*p* < 0.01 and *p* < 0.001, respectively), and the incident rate ratio between the two group was 1.28 (Table 4).

### 3.5. Evaluation of Baseline Pathogen Eradication

Wound microflora pathogens isolated at baseline are summarized in Table 2. The most prevalent identified baseline pathogen for both groups was *Staphylococcus aureus* (*S. aureus*), followed by *Pseudomonas aeruginosa* (*P. aeruginosa*) and *Escherichia coli* (*E. coli*), accounting respectively for more than 55%, 27%, and 11% of cases in each group. Statistical analyses revealed that the proportion of patients with of baseline pathogen eradication on day 12 was significantly higher in the study group A, compared to the control group B (15.1 ± 2.5 vs. 9.3 ± 6.1, *p* < 0.01, Table 3). Throughout the entire study, the pathogen eradication rate in the study group did not cease to significantly increase compared to the control group.

## 4. Discussion

Our study investigated the comparative application and clinical effectiveness of antibiotic-loaded bone cement with vacuum sailing drainage (VSD) in the treatment of neuropathic diabetic foot ulcers complicated by osteomyelitis. The conception of our study was designed to assess if individually adding these two different therapies to the standard care (which mainly includes systemic antibiotherapy combined with standard diabetic wound management) showcases the clinical benefits of ALBC in diabetic foot ulcers suggestive to osteomyelitis. As one of the leading causes of chronic diseases and limb loss worldwide, diabetes mellitus (DM) affects developing countries disproportionately as more than 80% of diabetes deaths occur in low- and middle-income countries [14, 17]. Diabetic foot (DF) is described by a decrease in pain and temperature sensation first and later by a decrease in vibratory sensitivity and superficial touch, resulting to the patient being unable to feel painful mechanical, chemical, or thermal stimuli in normal situations [18, 19]. Most diabetic foot infections (DFIs) occur with neuropathic ulcers, which serves as a point of entry for pathogens [16], with approximately 60% of DFUs infected on presentation [20].

Foot ulcerations in diabetic patients are a major health problem. The latter stages of complications are usually associated with serious morbidity and reduction of quality of life. By history and through clinical examination, diabetic foot ulcers fall into three categories: neuropathic, neuroischemic, and ischemic [6, 12, 21, 22]. Their management has been addressed in various ways, and this has led to the recommendation and implementation of various local or regional clinical practical guidelines [6]. Nevertheless, all these clinical practical guidelines tend to follow the same patterns that include foot evaluation, pharmacological therapy, offloading, wound dressing, negative pressure wound therapy, and education for patients and relatives [2, 6, 19].

Assessing the diagnosis of diabetic foot osteomyelitis (DFO) requires a strong correlation between the clinical, histologic, and imaging studies presented in the individual patient [23]. While it is worthy of note that differentiating DFO from Charcot neuroosteoarthropathy presents considerable challenges and requires strict evaluation of the patient [24], studies revealed that DFO may be present in up to 20% of mild to moderate infections and in 50% to 60% of severely infected wounds [25]. Even though the pathophysiologic mechanism of DFO has been well established in recent years, the consensus on its systematic treatment is still yet to be consolidated [23]. However, diabetic foot ulcers with additional infection such as osteomyelitis have also been shown to benefit from a targeted antibiotic regimen based on wound culture results, and the duration of treatment often depends on the severity of the underlying infection [2].

Therapies comprising of vacuum systems have been highly recommended to treat DFUs, especially due to their noninvasive clinical features in promoting wound healing and formation of granulation tissue [26]. Studies have showed the clinical evidence benefits of VSD in treating DFUs, as it is well tolerated and usually recommended [13, 26]. A systematic review by Huang et al. [26] showed VSD to be safe and effective for the treatment of DFUs in multivariable analysis when compared to conventional treatments. Results showed VSD to have better clinical outcomes, including short duration of therapy, low complication rate, short hospital LOS, and short complete wound closure and healing time. However, in the same paper, conventional therapies are found to be more efficacious than VSD in terms of wound size reduction [26]. Although tremendous progress in the clinical effectiveness and usage of systemic antibiotic for the treatment of DFO has been done, its efficacy had also been impaired by various factors [27, 28]. It is worthy of note that success of DFO treatment with local administration of antibiotics has been documented. In contrast to the administration of systemic antibiotics, the use of local antibiotics has an essential advantage of achieving high drug concentration in the infectious targeted area [27].

In this study, analysis of clinical outcomes showed that all patients in study group had a shorter healing time, 1 patient reported postoperative complication, and there were no reulceration episodes recorded within the 12 months of fellow-up period. Patients in control not only had a relative longer healing period but also recorded episodes of reulceration (2 patients) and postoperative complications (2 patients).

Diabetic foot ulcers are more inclined to bacterial infections that usually spread rapidly and lead to irreversible clinical outcomes [29]. Studies indicate that *Staphylococcus*, *Pseudomonas*, and *Escherichia* are among the most prevalent genera that isolated pathogens from diabetic foot ulcers [30–33]. Based on clinical practice guidelines available, there have been no sound evidence of preferential choice regarding the effectiveness of antibiotic's type [1]. The selection for the most appropriate and effective antibiotic therapy always requires to define the specific causative pathogens, especially as more than one bacteria are often found to be at the core of diabetic foot infection [29]. The empirical treatment is initially based on wound severity since culturing and profiling the antibiotic sensitivity of wound-associated microbes is time-consuming [31]. In our study population, the bacteriological analysis at baseline revealed predominance of one aerobic Gram-positive coccus (*S. aureus*) and of two aerobic Gram-negative bacilli (*P. aeruginosa* and *E. coli*). These results relatively corroborate findings from other investigations [31–35]. Although Gram-negative bacilli as a group were most prevalent bacteria isolated in our study, *S. aureus* was the most predominant isolate, accounting for 61.12% and 55.56% of all microorganisms in control and study group, respectively. These findings share similar trend with previous studies [34, 35]. Findings from recent studies have showed that aerobic Gram-negative bacilli, especially *P. aeruginosa* and *E. coli*, are reported among the most prevalent causative pathogens of DFU in hot climates areas and developing countries [31–33, 35, 36]. Although these findings are quite interesting, it is worthy of note that no studies have established any correlation with environmental or hygienic factors [37]. During our study, once the wound microbial cultures were confirmed, antibiotics were narrowed and tailored based on microbial species present on the cultures. Twelve days after the implementation of the specific antibiotherapy regimens in each group, the study group recorded a significantly higher number of patients with baseline pathogen eradication compared to the control. Based on the antibiotic sensitivity data, the antibiotics loaded were the vancomycin, gentamicin, and cefoperazone (*broad-spectrum cephalosporins*) plus sulbactam (*extended-spectrum β-lactam*). Numerous studies showed that topical antibiotics have been increasingly used for prevention and treatment of different types of infections [38, 39]. In various studies, antimicrobial sensitivity test data revealed that isolates from *S. aureus*, *P. aeruginosa*, and *E. coli* are sensitive to different classes of antibiotics; this might be due to the severity or to the polymicrobial status of the infection. DFIs from these isolate organisms have been treated by various antibiotics regimens as they showed sensitivity to penicillins of moderate to broad spectrum (piperacillin/tazobactam, amoxicillin/clavulanic acid) [32, 33], broad-spectrum cephalosporins plus *β*-lactams (cefoperazone/sulbactam) [13], and aminoglycosides (gentamicin) [16] for *P. aeruginosa*; moderate-spectrum cephalosporins (cefoxitin) [33], glycopeptides (vancomycin) [13, 33], aminoglycosides (gentamicin) [16, 40], and other antibacterials (nitrofurantoin) [33] for *S. aureus*; and aminoglycosides (amikacin, gentamicin) [13, 16, 33, 35] and other antibacterials (nitrofurantoin) [33] for *E. coli*.

Most of diabetic foot patients experience neuropathic pain as it was thought to be associated with peripheral nerve problems such as neuropathy caused by DM, but sometimes, neuropathic pain can be contrasted to nociceptive pain, and every pain located in the lower limbs of patients with diabetic neuropathy is not necessarily neuropathic [41]. Although its management has evolved throughout the years, an overall analysis of current clinical practices guidelines found its evidence to be controversial [1]. Nevertheless, within the diabetic foot population, pain still remains an essential pathological condition through which physician assesses preoperative medical severity and postoperative outcomes [14]. In the current study, data related to pain intensity showed that with an average reported pain score (NRS) of 3.3 (control group) and 1.4 (study group), the control group's average scores were 2.35 times higher than the average study group score. As this pain assessment was only done postoperatively, this significant difference could be subjective to the difference of clinical effectiveness between the two treatment methods. For a diabetic foot patient, the ability to evaluate the patient pain is of great interest as it contributes to a better compliance and quick recovery.

There are several helpful indicators of clinical severity and resources, and hospital length of stay (LOS) is one of them especially in the area of diabetic foot diseases [14]. Assessing patient's rational LOS gives us the possibility to evaluate the degree and quality of hospital care that the patient cannot receive elsewhere [42]. The mean hospital LOS in the studied patients was recorded to be 29 and 25 days in the control group and study group, respectively. Analysis of data suggested that patients from control group did spend an overall mean of 1.2 days longer than those from the study group. Even though there was no statistical difference between the two group, it is worth noting that the need for hospitalization is a parameter which might affect the mood of diabetic patients [14]. Hospital LOS has also significantly been associated with number of variables such as the time and type of admission, place of residence, and quality of care [43]. As all patients were admitted into the same department, on same wards, and were treated by the same medical staff, this hospital LOS could only be subjective to the quality of therapy.

One of the key issues for the therapy of DFU is the prolonged wound healing time, which may have resulted from traditional treatment [21]. The etiological and anatomical heterogeneity of diabetic foot ulcers have made challenging the assessment of wound healing and prediction of healing time [11]. Data have shown that one-third of neuropathic ulcer completely healed after 20 weeks of good care [44], and the wound size reduction within the first month of treatment can be a prognostic factor of healing rate [45]. A validated equation established by Zimny et al. [21] found to be reliable is used as a prognostic assessment tool of healing time in diabetic neuropathic foot ulcers. In the current study, both groups recorded a significant decrease of wound area after 12 weeks. The study group had a period course of healing time (incident rate ratio) of 1.2 times faster compared to the control group (101.7 vs. 79.4). According to the wound radius reduction calculated with the equation, the study group experienced a significant speedy course healing time compared to the control group (0.049 vs. 0.037). Findings from previous studies assessing the healing time in DFOs showed that with conventional treatment, the mean of healing time can fall within the interval of 181-267 days [46, 47]. In the current study, with administration of topical antibiotics, data analysis showed that this time could significantly decrease by more than half. Although there might be various factors to be taken in consideration, based on healing times and calculated mean wound radius reductions in both groups, the impact of wound healing of the ALBC is found statistically more efficient than VSD's. These findings also revealed the effectiveness of ALBC compared to other methods of therapy.

The need for more high-quality clinical evidence of ALBC efficacy, especially with large randomized controlled trials, suggested that economically, there is no evidence that ALBC is cost-effective to prevent or treat musculoskeletal infection (MSKI) such as osteomyelitis [48]. Cost-effectiveness analyses of ALBC vary across markets due to factors such as surgeons' experience, price of sensitive antibiotic, choice of biomedical material used, and severity of the infections. Nevertheless, the longer a wound takes time for a complete healing, costly will be the management of DFOs due to its direct impact on human and financial resources. With an estimated annual cost of US $8659 per patient, diabetes foot care poses an increasing socioeconomic burden for the public health [49]. The cost-effectiveness analyses of ALBC have mostly been done in the knee, hip, and shoulder arthroplasty studies [50–54]. Moreover, in order to achieve the standardization of ALBC use, these studies have investigated factors such as cement/antibiotic mix models [50], quantity of antibiotics loaded [50, 51], and nature of bone cement [52–54]. According to the findings, manually mixed models and dual antibiotic-loaded bone cement were more cost-effective than premixed models and single antibiotic-loaded bone cement, respectively [50, 51]. However, in terms of nature of bone cement, findings showed that ALBC is costlier than plain bone cement (PBC) and brings along some unnecessary financial burden healthcare systems, which might already struggle to maintain high quality of care [52, 54]. In this current study, data analyses showed that the ALBC attributed to study group patients was significantly more cost-effective than the VSD allocated to control group patients.

Managing complex diabetic foot complications such as osteomyelitis is a process that requires the integration of multidisciplinary members' team. This specialist team frequently but not invariably comprises a diabetologist, podiatrist, microbiologist, tissue viability nurse, orthopedic surgeon, and vascular surgeon with a thorough understanding of foot function [2]. This team must first and foremost act with mutual respect and understanding [6]. In addition, the delivery of an effective service facilitated by input from the multidisciplinary team has demonstrated significant benefit in reducing incidence of both minor and major amputations [55].

Overall, it is worth noting that while few studies [16, 40, 56] have proven the benefit of antibiotic-loaded bone cement in clinical management of diabetic foot osteomyelitis, some investigations such as Chatzipapas et al.'s study [57] have not find the adjunctive local antibiotic therapy to improve the outcomes in surgically treated DFO. However, compared to our study, this can be explained by inclusion of patients with Wagner grades of I and II in the final analysis. Moreover, the ulcer severity of such patients could have been a potential influential factor in the final statistical analysis and clinical outcomes, especially that they represent 36% patients of the study population in that study. Thus, as taken together, these studies seem to present the benefit of local antibiotic systems in diabetic foot osteomyelitis management as inconclusive, larger, and prospective trials are still needed to further assess these treatment options. And if such trials can really show the pros and cons of local antibiotic therapy regarding benefits of patients in overall or for any target subgroups, the knowledge acquired will be helpful to address in diabetic clinical settings.

### 4.1. Limitations

Although we attempted a well-designed study, some limitations inherent were inevitably found in our study. The primary limitation of this study is its small sample size based on its period study. Secondary, comorbidities were not recorded, and their analysis was not conducted to assess their impact on the final clinical outcomes. Moreover, an extensive wound core microbiome analysis was not carried out to assess its association with the wound severity. Further larger studies, with longer follow-up and different types of ulcers while assessing the clinical effectiveness the ALBC, are needed to strengthen these conclusions. Despite these limitations, the current study provides a sustainable and effective clinical value of ALBC in the treatment of diabetic foot ulcerations.

## 5. Conclusion

This randomized controlled study supports the clinical application and efficacy of antibiotic-loaded bone cement in the treatment of neuropathic diabetic foot ulcer complicated with osteomyelitis. This technique reduces the number of operations or dressing changes and reduces doctors' burden, shortens the length of hospital stay, and therefore reduces the medical cost of the patient and makes it easy for outpatient follow-up. However, there is still need for more evidence studies, and the technology is worthy of popularization and application as it is a promising option in the treatment of DFUs.

## Figures and Tables

**Table 1:** Study population demographic characteristics.

	No. of patients	Gender (*n*)	Mean age (years)	HbA1c (%)	Type of diabetes	BMI (kg/m^2^)	ABI	Ulceration site	Wagner grade
		M/F			1/2			F/M/H	III/IV
Control group	18	11/7	45 ± 6.3	7.4 ± 1.3	5/13	28.3 ± 0.5	1.0 ± 0.2	7/6/5	10/8
Study group	18	13/5	48 ± 5.1	7.9 ± 0.4	3/15	27.9 ± 0.7	1.0 ± 0.1	11/5/2	11/7
*p* value		>0.05	>0.05	>0.05	>0.05	>0.05	>0.05	>0.05	>0.05

Data presented as mean ± standard deviation; gender: M, male; F, Female; HbA1c, glycated hemoglobin; BMI, body mass index; ABI, ankle brachial index; ulceration site: F, forefoot; M, midfoot; H, hindfoot.

**Table 2:** Prevalent pathogens isolated from diabetic wound at baseline.

Bacterial culture	Control group, *n* (%)	Study group, *n* (%)
*Staphylococcus aureus*	11 (61.12%)	10 (55.56%)
*Pseudomonas aeruginosa*	5 (27.77%)	6 (33.33%)
*Escherichia coli*	2 (11.11%)	2 (11.11%)
Total	18 (100%)	18 (100%)

**Table 3:** Comparison of patients postoperative clinical outcomes.

Comparison index	Control group (n = 18)	Study group (n = 18)	*p* value
Baseline pathogen eradication (No. of patients)	9.3 ± 6.1	15.1 ± 2.5	<0.01
NRS pain	3.3 ± 1.4	1.4 ± 0.9	<0.01
Number of dressing change (mean, during hospital)	5.6 ± 3.4	3.5 ± 1.2	<0.01
Mean length of hospital stay (day)	29.0 ± 2.0	25.0 ± 2.0	>0.05
Average hospital cost ($)	4100 ± 800	3700 ± 460	<0.05
Complications	2	1	<0.05
Infection recurrence	2	0	<0.01

Data presented as mean ± standard deviation; NRS pain, numeric rating scale for pain; $, US dollar; baseline pathogen eradication assessed on day 12.

**Table 4:** Wound parameters and healing time.

Comparison index	Control group (*n* = 18)	*p* value	Study group (*n* = 18)	*p* value	IRR
Wound surface at day 0 (cm^2^)	18.0 ± 10.5	<0.05	17.5 ± 11.0	0.05	
Wound surface at day 84 (cm^2^)	3.2 ± 0.5		2.1 ± 0.5		
Wound radius at day 0 (cm)	3.75 ± 0.7	<0.001	3.36 ± 0.6	<0.001	
Wound radius at day 84 (cm)	0.77 ± 0.34		0.62 ± 0.22		
Wound radius reduction (cm)	0.037 (95% CI, 0.033–0.041)	—	0.049 (95% CI, 0.043–0.057)^∗^	—	—
Wound healing time (days)	101.7 (95% CI, 93–110)	—	79.4 (95% CI, 71–90)^**#**^	—	1.28

Data presented as mean ± standard deviation; IRR, incident rate ratio (IRR is the average healing time between the two groups). ^∗^*p* < 0.01 for healing time in the study group vs. control group. ^#^*p* < 0.001 for mean wound radius reduction in the study group vs. control group.

## Data Availability

No data were used to support this study.

## Abbreviations

ABI: Ankle brachial index
ALBC: Antibiotic-loaded bone cement
DM: Diabetes mellitus
DFI: Diabetic foot infection
DFU: Diabetic foot ulcer
DFO: Diabetic foot osteomyelitis
LOS: Length of stay
NRS: Numeric rating scale
VSD: Vacuum sealing drainage.

## References

[1] Pérez-Panero A. J., Ruiz-Muñoz M., Cuesta-Vargas A. I., Gónzalez-Sánchez M. (2019). Prevention, assessment, diagnosis and management of diabetic foot based on clinical practice guidelines: a systematic review. *Medicine*.

[2] Lim J. Z., Ng N. S., Thomas C. (2017). Prevention and treatment of diabetic foot ulcers. *Journal of the Royal Society of Medicine*.

[3] Lepäntalo M., Apelqvist J., Setacci C. (2011). Chapter V: diabetic foot. *European Journal of Vascular and Endovascular Surgery*.

[4] Nunan R., Harding K. G., Martin P. (2014). Clinical challenges of chronic wounds: searching for an optimal animal model to recapitulate their complexity. *Disease Models & Mechanisms*.

[5] Reiber G. E., Lipsky B. A., Gibbons G. W. (1998). The burden of diabetic foot ulcers. *American Journal of Surgery*.

[6] Schaper N. C., Netten J. J., Apelqvist J. (2020). Practical guidelines on the prevention and management of the diabetic foot (IWGDF 2019 update). *Diabetes/Metabolism Research and Reviews*.

[7] Singh R., Kishore L., Kaur N. (2014). Diabetic peripheral neuropathy: current perspective and future directions. *Pharmacological Research*.

[8] Aragón-Sánchez J., Quintana-Marrero Y., Lázaro-Martínez J. L. (2009). Necrotizing soft tissue infections in the feet of patients with diabetes: outcome of surgical treatment and factors associated with limb loss and mortality. *The International Journal of Lower Extremity Wounds*.

[9] Lavery L. A., Armstrong D. G., Murdoch D. P., Peters E. J. G., Lipsky B. A. (2007). Validation of the Infectious Diseases Society of America’s diabetic foot infection classification system. *Clinical Infectious Diseases*.

[10] Láazaro-Martíınez J. L., Aragón-Sánchez J., García-Morales E. (2014). Antibiotics versus conservative surgery for treating diabetic foot osteomyelitis: a randomized comparative trial. *Diabetes Care*.

[11] Zimmy S., Pfohl M. (2005). Healing times and prediction of wound healing in neuropathic diabetic foot ulcers: a prospective study. *Experimental and Clinical Endocrinology & Diabetes*.

[12] Zimny S., Schatz H., Pfohl M. (2003). The effects of applied felted foam on wound healing and healing times in the therapy of neuropathic diabetic foot ulcers. *Diabetic Medicine*.

[13] Liu X., Liang J., Zao J. (2016). Vacuum sealing drainage treatment combined with antibiotic-impregnated bone cement for treatment of soft tissue defects and infection. *Medical Science Monitor*.

[14] Jiang L. P., Mendame Eyha R. E. (2020). Effectiveness of a collaborative nursing care model for the treatment of patients with diabetic foot disease by transverse tibial bone transport technique: a pilot study. *Journal of Perianesthesia Nursing*.

[15] Li R.-G., Yu B., Wang G. (2012). Sequential therapy of vacuum sealing drainage and free-flap transplantation for children with extensive soft-tissue defects below the knee in the extremities. *Injury*.

[16] Niazi N. S., Drampalos E., Morrissey N., Jahangir N., Wee A., Pillai A. (2019). Adjuvant antibiotic loaded bio composite in the management of diabetic foot osteomyelitis — a multicentre study. *The Foot*.

[17] International Diabetes Federation (2017). *IDF Diabetes Atlas*.

[18] Cruciani M., Lipsky B. A., Mengoli C., de Lalla F. (2013). Granulocyte-colony stimulating factors as adjunctive therapy for diabetic foot infections. *Cochrane Database of Systematic Reviews*.

[19] Jones N. J., Harding K. 2015 International Working Group on the Diabetic Foot Guidance on the prevention and management of foot problems in diabetes. *International Wound Journal*.

[20] Prompers L., Huijberts M., Apelqvist J. (2007). High prevalence of ischaemia, infection and serious comorbidity in patients with diabetic foot disease in Europe. Baseline results from the Eurodiale study. *Diabetologia*.

[21] Zimny S., Schatz H., Pfohl M. (2002). Determinants and estimation of healing times in diabetic foot ulcers. *Journal of Diabetes and its Complications*.

[22] Yotsu R. R., Pham N. M., Oe M. (2014). Comparison of characteristics and healing course of diabetic foot ulcers by etiological classification: neuropathic, ischemic, and neuro-ischemic type. *Journal of Diabetes and its Complications*.

[23] Hingorani A., LaMuraglia G. M., Henke P. (2016). The management of diabetic foot: a clinical practice guideline by the Society for Vascular Surgery in collaboration with the American Podiatric Medical Association and the Society for Vascular Medicine. *Journal of Vascular Surgery*.

[24] Ertugrul B. M., Lipsky B. A., Savk O. (2013). Osteomyelitis or Charcot neuro-osteoarthropathy? Differentiating these disorders in diabetic patients with a foot problem. *Diabet Foot Ankle*.

[25] Lipsky B. A. (1997). Osteomyelitis of the foot in diabetic patients. *Clinical Infectious Diseases*.

[26] Huang Q., Wang J.-T., Gu H.-C., Cao G., Cao J.-C. (2019). Comparison of vacuum sealing drainage and traditional therapy for treatment of diabetic foot ulcers: a meta-analysis. *The Journal of Foot and Ankle Surgery*.

[27] Markakis K., Faris A. R., Sharaf H., Faris B., Rees S., Bowling F. L. (2018). Local antibiotic delivery systems: current and future applications for diabetic foot infections. *The International Journal of Lower Extremity Wounds*.

[28] Morley R., Lopez F., Webb F. (2016). Calcium sulphate as a drug delivery system in a deep diabetic foot infection. *Foot (Edinburgh, Scotland)*.

[29] Lipsky B. A., Berendt A. R., Cornia P. B. (2012). 2012 Infectious Diseases Society of America clinical practice guideline for the diagnosis and treatment of diabetic foot infections. *Clinical Infectious Diseases*.

[30] Bowler P. G., Duerden B. I., Armstrong D. G. (2001). Wound microbiology and associated approaches to wound management. *Clinical Microbiology Reviews*.

[31] Jnana A., Muthuraman V., Varghese V. K. (2020). Microbial community distribution and core microbiome in successive wound grades of individuals with diabetic foot ulcers. *Applied and Environmental Microbiology*.

[32] Tiwari S., Pratyush D. D., Dwivedi A., Gupta S. K., Rai M., Singh S. K. (2012). Microbiological and clinical characteristics of diabetic foot infections in northern India. *Journal of Infection in Developing Countries*.

[33] Mutonga D. M., Mureithi M. W., Ngugi N. N., Otieno F. C. F. (2019). Bacterial isolation and antibiotic susceptibility from diabetic foot ulcers in Kenya using microbiological tests and comparison with RT-PCR in detection of S. aureus and MRSA. *BMC Research Notes*.

[34] Citron D. M., Goldstein E. J. C., Merriam C. V., Lipsky B. A., Abramson M. A. (2007). Bacteriology of moderate-to-severe diabetic foot infections and in vitro activity of antimicrobial agents. *Journal of Clinical Microbiology*.

[35] Djahmi N., Messad N., Nedjai S. (2013). Molecular epidemiology of Staphylococcus aureus strains isolated from inpatients with infected diabetic foot ulcers in an Algerian University Hospital. *Clinical Microbiology and Infection*.

[36] Chalya P. L., Mabula J. B., Dass R. M. (2011). Surgical management of diabetic foot ulcers: a Tanzanian University teaching hospital experience. *BMC Research Notes*.

[37] Spichler A., Hurwitz B. L., Armstrong D. G., Lipsky B. A. (2015). Microbiology of diabetic foot infections: from Louis Pasteur to ‘crime scene investigation’. *BMC Medicine*.

[38] Lipsky B. A., Hoey C. (2009). Topical antimicrobial therapy for treating chronic wounds. *Clinical Infectious Diseases*.

[39] McHugh S. M., Collins C. J., Corrigan M. A., Hill A. D. K., Humphreys H. (2011). The role of topical antibiotics used as prophylaxis in surgical site infection prevention. *The Journal of Antimicrobial Chemotherapy*.

[40] Lipsky B. A., Kuss M., Edmonds M., Reyzelman A., Sigal F. (2012). Topical application of a gentamicin-collagen sponge combined with systemic antibiotic therapy for the treatment of diabetic foot infections of moderate severity: a randomized, controlled, multicenter clinical trial. *Journal of the American Podiatric Medical Association*.

[41] Bouhassira D. (2019). Neuropathic pain: definition, assessment and epidemiology. *Revue Neurologique (Paris)*.

[42] Clarke A. (2002). Length of in-hospital stay and its relationship to quality of care. *Quality & Safety in Health Care*.

[43] Baniasadi T., Kahnouji K., Davaridolatabadi N., Hosseini Teshnizi S. (2019). Factors affecting length of stay in Children Hospital in Southern Iran. *BMC Health Services Research*.

[44] Margolis D. J., Kantor J., Berlin J. A. (1999). Healing of diabetic neuropathic foot ulcers receiving standard treatment. A meta-analysis. *Diabetes Care*.

[45] Pham H., Falanga V., Sabolonski M. L. (2000). Healing rate measurement can predict complete wound healing rate in chronic diabetic foot ulceration. *Diabetologia*.

[46] Milne T. E., Schoen D. E., Bower V. M. (2013). Healing times of diabetic foot ulcers: investigating the influence of infection and peripheral arterial disease. *J Diabet Foot Complications*.

[47] Lavery L. A., Armstrong D. G., Peters E. J. G., Lipsky B. A. (2007). Probe-to-bone test for diagnosing diabetic foot osteomyelitis. *Diabetes Care*.

[48] Schwarz E. M., McLaren A. C., Sculco T. P. (2021). Adjuvant antibiotic-loaded bone cement: concerns with current use and research to make it work. *Journal of Orthopaedic Research*.

[49] Raghav A., Khan Z. A., Labala R. K., Ahmad J., Noor S., Mishra B. K. (2018). Financial burden of diabetic foot ulcers to world: a progressive topic to discuss always. *Therapeutic Advances in Endocrinology and Metabolism*.

[50] Gutowski C. J., Zmistowski B. M., Clyde C. T., Parvizi J. (2014). The economics of using prophylactic antibiotic-loaded bone cement in total knee replacement. *Bone Joint J*.

[51] Sanz-Ruiz P., Matas-Diez J. A., Villanueva-Martínez M., Santos-Vaquinha Blanco A. D., Vaquero J. (2020). Is dual antibiotic-loaded bone cement more effective and cost-efficient than a single antibiotic-loaded bone cement to reduce the risk of prosthetic joint infection in aseptic revision knee arthroplasty?. *The Journal of Arthroplasty*.

[52] Hoskins T., Shah J. K., Patel J. (2020). The cost-effectiveness of antibiotic-loaded bone cement versus plain bone cement following total and partial knee and hip arthroplasty. *Journal of Orthopaedics*.

[53] Jameson S. S., Asaad A., Diament M. (2019). Antibiotic-loaded bone cement is associated with a lower risk of revision following primary cemented total knee arthroplasty: an analysis of 731, 214 cases using National Joint Registry data. *Bone Joint J*.

[54] Nowinski R. J., Gillespie R. J., Shishani Y., Cohen B., Walch G., Gobezie R. (2012). Antibiotic-loaded bone cement reduces deep infection rates for primary reverse total shoulder arthroplasty: a retrospective, cohort study of 501 shoulders. *Journal of Shoulder and Elbow Surgery*.

[55] Krishnan S., Nash F., Baker N., Fowler D., Rayman G. (2008). Reduction in diabetic amputations over 11 years in a defined UK population: benefits of multi-disciplinary team work and continuous prospective audit. *Diabetes Care*.

[56] Varga M., Sixta B., Bem R., Matia I., Jirkovska A., Adamec M. (2014). Application of gentamicin-collagen sponge shortened wound healing time after minor amputations in diabetic patients—a prospective, randomised trial. *Archives of Medical Science*.

[57] Chatzipapas C., Kougioumtzis I. E., Karaglani M. (2020). Local antibiotic delivery systems in the surgical treatment of diabetic foot osteomyelitis: again, no benefit?. *The International Journal of Lower Extremity Wounds*.

